# Feline Lower-Lip Apocrine Sweat Gland Adenocarcinoma with Mandibular Nodal Metastasis: A Case Report

**DOI:** 10.3390/vetsci13070606

**Published:** 2026-06-23

**Authors:** Yongwon Park, Ajin Lee, Jeonghoon Jang, Hwi-Yool Kim

**Affiliations:** 1 24Soom Animal Hospital, 715 Gwonseon-ro, Gwonseon-gu, Suwon 16568, Republic of Korea; spid19@naver.com (Y.P.);; 2 Department of Veterinary Surgery, College of Veterinary Medicine, Konkuk University, 120 Neungdong-ro, Gwangjin-gu, Seoul 05029, Republic of Korea

**Keywords:** cytology, histopathology, immunohistochemistry, adnexal tumor, salivary gland differential, lymphatic invasion

## Abstract

Apocrine sweat gland adenocarcinoma is an uncommon feline adnexal neoplasm, and lower-lip involvement is rarely documented. A 14-year-old spayed female Siamese cat was evaluated for a firm 1.3 cm right lower-lip mass at a documented hemorrhagic lesion site. Approximately 1 year earlier, the lesion was fluctuant and superficially erosive to ulcerative; fine-needle aspiration yielded bloody fluid with cytologically nondiagnostic material. The lesion reportedly subsided after empirical treatment, but reassessment, tissue diagnosis, and objective confirmation of resolution were not performed. Cytology of the later same-site mass supported malignant epithelial neoplasia. Contrast-enhanced computed tomography (CT) identified a 12.5 × 13.2 × 16.6 mm rim-enhancing mass without mandibular invasion or distant metastasis; the ipsilateral mandibular lymph node was mildly thickened. Histopathology supported a diagnosis of lower-lip apocrine sweat gland adenocarcinoma with lymphovascular tumor emboli, lymphatic invasion, and nodal metastasis. The diagnosis was supported by superficial dermal/subcutaneous location, adjacent adnexal structures, skeletal-muscle separation from nearby minor salivary glands, epithelial immunoreactivity, periodic acid-chiff (PAS) and Alcian blue findings without distinct intratumoral mucin/mucopolysaccharide-rich material. The cat died without necropsy on postoperative day 87. This case supports tissue-based diagnosis and regional lymph node assessment for suspicious feline lower-lip glandular epithelial lesions.

## Simple Summary

Apocrine sweat gland adenocarcinoma is an uncommon feline skin adnexal carcinoma, and lower-lip involvement has been reported infrequently. This report describes a 14-year-old spayed female Siamese cat with lower-lip apocrine sweat gland adenocarcinoma and mandibular lymph node metastasis. Approximately 1 year before diagnosis, the cat had a fluctuant hemorrhagic lesion at the same lower-lip site with a small superficial erosive to ulcerative focus; fine-needle aspiration yielded bloody fluid with cytologically nondiagnostic material. Although the lesion reportedly subsided after empirical treatment, scheduled reassessment and complete clinical resolution were not documented. A firm mass was later identified at the same site, and cytology supported malignant epithelial neoplasia. Histopathology of the excised mass and ipsilateral mandibular lymph node supported a diagnosis of apocrine sweat gland adenocarcinoma with lymphatic invasion and nodal metastasis. The diagnosis was supported by superficial cutaneous location, association with cutaneous adnexal structures, lack of continuity with nearby minor salivary glands, epithelial immunoreactivity, and ancillary histochemical findings. This case illustrates the risk of underestimating a hemorrhagic, superficially eroded lower-lip lesion when cytology is nondiagnostic and objective resolution is not documented. Lower-lip lesions with persistent or subsequent same-site clinical concern may require tissue-based diagnosis and regional lymph node assessment.

## 1. Introduction

Feline apocrine sweat gland adenocarcinoma (ASGAC) is an uncommon malignant adnexal neoplasm, and the veterinary literature remains limited to small clinicopathologic series and isolated case reports [1,2,3,4,5,6]. Reported feline cases have arisen at multiple cutaneous sites, including the head, neck, axilla, trunk, and limbs, and can show diverse tubular, ductal, cystic, and papillary histologic patterns [2,4,5,6].

Lower-lip involvement appears particularly uncommon. A lower-lip apocrine ductal carcinoma was reported in a cat in 2011 [7], and a more recent rostral mandibular case documented recurrent ASGAC with mandibular nodal metastasis managed by multimodal therapy [8]. Additional isolated feline ASGAC cases have also been reported at other cutaneous sites [9].

Because the feline lower lip contains both cutaneous adnexal and minor salivary glandular structures, malignant glandular tumors in this region require careful interpretation in relation to anatomic topography, histomorphology, and ancillary findings. Minor salivary gland adenocarcinoma and salivary duct carcinoma are also recognized in cats and may present as labial or buccal masses with overlapping glandular epithelial morphology [10,11,12,13,14,15,16].

The present report describes a feline lower-lip ASGAC with mandibular nodal metastasis that was diagnosed using integrated clinical, topographic, histopathologic, histochemical, and immunohistochemical findings. The case occurred at the site of a previously documented hemorrhagic, superficially eroded, cytologically nondiagnostic lower-lip lesion that reportedly subsided after empirical treatment but was not objectively documented to have completely resolved. This report highlights the diagnostic and staging value of tissue-based diagnosis and regional lymph node assessment in suspicious same-site feline lower-lip glandular epithelial lesions.

## 2. Case Description

**Figure 1:**
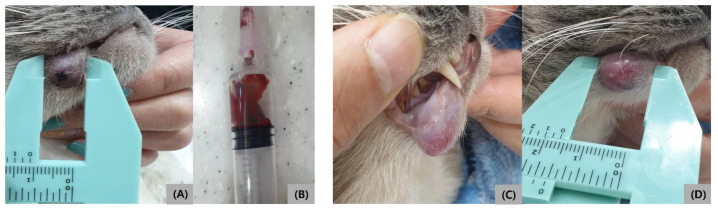
Clinical appearance of the lesion at the earlier and later same-site presentations. (**A**) Fluctuant right lower-lip lesion measuring approximately 1 cm at the earlier presentation in February 2023; a small superficial erosive to ulcerative focus is appreciable on the lesion surface. (**B**) Bloody aspirate obtained from the earlier lesion. (**C**) Firm right lower-lip mass identified at the same site approximately 1 year later, measuring about 1.3 cm with a caliper. (**D**) Mucosal aspect of the later same-site mass.

**Figure 2:**
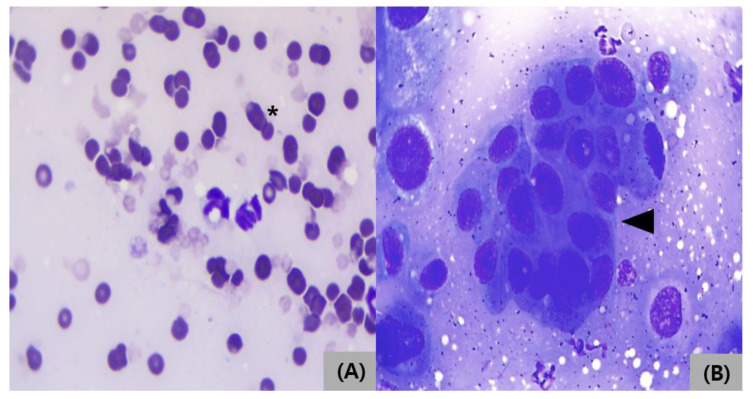
Cytology of the lesion at the earlier and later same-site presentations. (**A**) Smear from the earlier aspirate showing low cellularity and prominent blood contamination (asterisk), consistent with a nondiagnostic sample (Diff-Quik stain; original magnification ×400). (**B**) Smear from the later same-site mass showing cohesive atypical epithelial cells (arrowhead) with anisokaryosis and features suspicious for malignant epithelial neoplasia (Diff-Quik stain; original magnification ×400; archival image).

A 14-year-old spayed female Siamese cat was presented for evaluation of a firm right lower-lip mass at a site where a lesion had been documented previously. According to the medical record from February 2023, the earlier lesion was a fluctuant lower-lip lesion measuring approximately 1 cm at the same site. On retrospective review, a small superficial erosive to ulcerative focus was appreciable on the lesion surface (Figure 1A). Fine-needle aspiration (FNA) with a 19-gauge needle yielded bloody fluid, and smear evaluation was reported as non-specific/nondiagnostic. No biopsy, repeat cytologic sampling, or scheduled reassessment was performed at that time. Empirical oral medication, consisting of cephalexin (30 mg/kg, per os [PO]), famotidine (0.5 mg/kg, PO), and bromelain (0.5 tablet, PO), together with local wound care, was prescribed for three days. The owner later reported that the lesion had subsided after treatment; however, complete clinical resolution was not objectively documented. Because the earlier lesion was not histologically characterized and was not followed by objective reassessment, its relationship to the later same-site mass could not be determined retrospectively. Representative gross and cytologic findings from the earlier presentation are shown in Figure 1A,B and Figure 2A.

Approximately 1 year later, a firm, smoothly contoured mass measuring approximately 1.3 cm was identified at the same right lower-lip site (Figure 1C,D). Cytologic examination revealed cohesive epithelial cells with marked anisokaryosis, supporting malignant epithelial neoplasia (Figure 2B).

**Figure 3:**
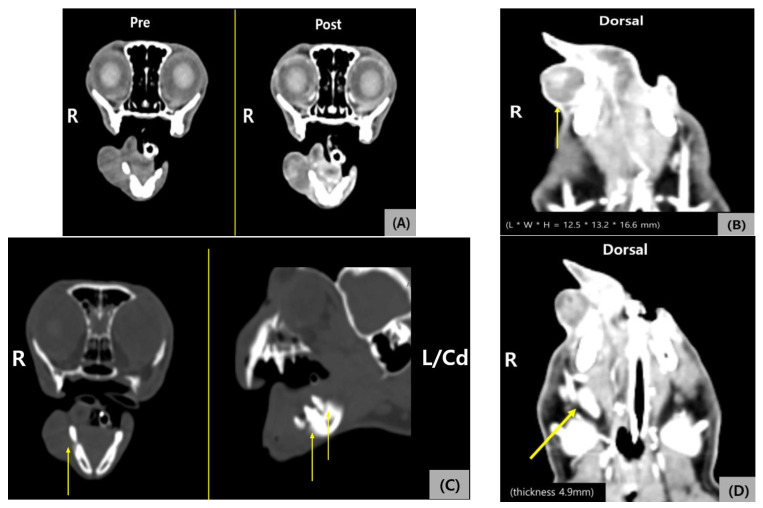
CT findings of the lower-lip mass and ipsilateral mandibular lymph node at the later same-site presentation. (**A**) Transverse pre- and post-contrast soft-tissue window images showing a well-defined rim-enhancing mass in the right lower-lip/mandibular region. (**B**) Post-contrast dorsal reconstructed image showing the extent of the mass (12.5 × 13.2 × 16.6 mm). (**C**) Pre-contrast bone-window image showing no obvious invasion of the adjacent mandible. (**D**) Post-contrast image showing mild thickening and enhancement of the ipsilateral mandibular lymph node (4.9 mm in thickness). Yellow arrows indicate the primary mass in panel (**B**), the adjacent mandibular bone region evaluated for invasion in panel (**C**), and the ipsilateral mandibular lymph node in panel (**D**).

Contrast-enhanced computed tomography (CT) encompassing the head and neck, thorax, and abdomen was performed under general anesthesia for staging and surgical planning using a 16-slice Toshiba CT scanner before and after intravenous administration of iohexol at 2.3 mL/kg. Images were reconstructed at a 1.0-mm slice thickness. A rim-enhancing mass with a well-defined margin was identified in the right mandibular/lower-lip region, measuring 12.5 × 13.2 × 16.6 mm. A small amount of internal enhancement was present, but there was no obvious mandibular bone invasion. No definite pulmonary, abdominal, or other distant metastatic lesion was identified on CT. The right mandibular lymph node showed mild contrast enhancement and thickening, with a measured thickness of 4.9 mm; other visualized mandibular lymph nodes were not enlarged. Radiologic interpretation of the ipsilateral node remained indeterminate, and the lymph node was considered compatible with reactive/inflammatory change versus possible early metastasis (Figure 3A–D).

Five days after the later same-site presentation and CT-based staging, the cat was prepared for aseptic facial/oral surgery under general inhalational anesthesia and standard perioperative monitoring. The lower-lip mass was locally excised together with the ipsilateral mandibular lymph node. Closure was achieved using local tissue apposition without a complex reconstructive procedure, and the lip mass and lymph node were submitted as separate specimens. The postoperative course was complicated by partial wound necrosis/dehiscence that required repeated rechecks, local wound care, and intermittent debridement, but progressive contraction and healing were documented during serial follow-up examinations through postoperative day (POD) 35.

The excised lip mass and mandibular lymph node were fixed in 10% neutral-buffered formalin, routinely processed, paraffin embedded, sectioned, and stained with hematoxylin and eosin. The specimens were evaluated by one diagnostic veterinary pathologist. Histopathologic examination yielded a diagnosis of apocrine adenocarcinoma with lymphatic invasion. The lesion was interpreted as a malignant glandular epithelial tumor of sweat-gland/adnexal origin within the lip skin.

**Figure 4:**
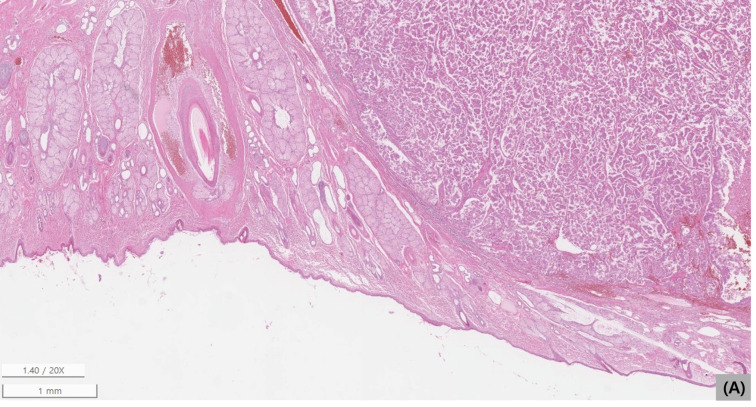

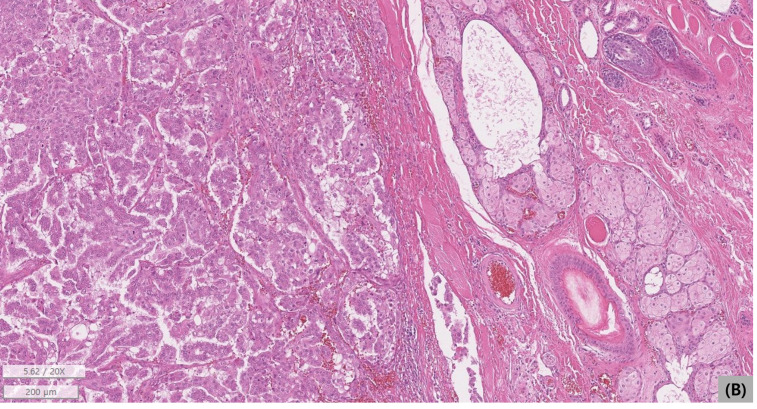

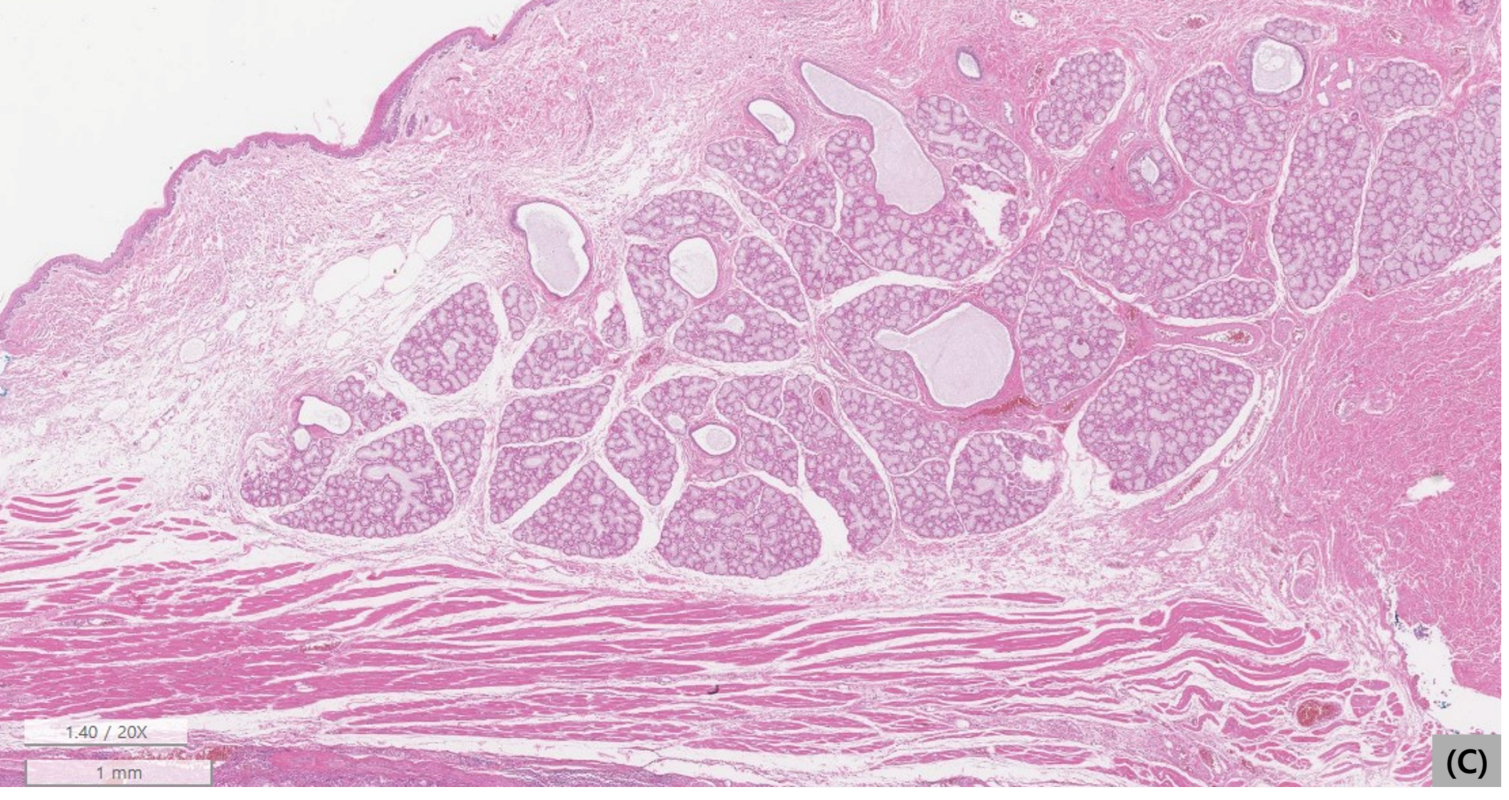
Histopathologic topography of the primary lower-lip tumor and adjacent glandular structures. (**A**) Low-power H&E section showing the neoplastic nodule within the superficial lip skin/dermal–subcutaneous compartment and its relationship to adjacent non-neoplastic cutaneous adnexal structures (scale bar = 1 mm). (**B**) Higher-power H&E section showing neoplastic glandular epithelial proliferation adjacent to non-neoplastic cutaneous adnexal structures, including sebaceous glands, tactile hair follicles, and sweat glands (scale bar = 200 µm). (**C**) Low-power H&E section showing nearby minor salivary gland tissue in the mucosal-side stroma, separated from the neoplastic region by skeletal muscle, without histologic continuity with the tumor (scale bar = 1 mm).

Topographic assessment showed that the neoplastic nodule was located within the superficial lip skin/dermal-subcutaneous compartment and was associated with adjacent non-neoplastic cutaneous adnexal structures (Figure 4A). At higher magnification, the tumor was adjacent to sebaceous glands, tactile hair follicles, and sweat glands (Figure 4B). Nearby minor salivary gland tissue was present within the mucosal-side stroma, but it was separated from the neoplastic region by skeletal muscle, and no histologic continuity between the tumor and salivary gland parenchyma was identified (Figure 4C). These topographic findings, together with the tubular/glandular architecture described below, supported a cutaneous adnexal/apocrine origin. Distinct apical blebbing/decapitation secretion was not clearly identified; however, the tumor was poorly differentiated and markedly atypical, limiting reliance on this single feature. Overall, the anatomic distribution and histologic architecture favored ASGAC over a minor salivary gland carcinoma.

**Figure 5:**
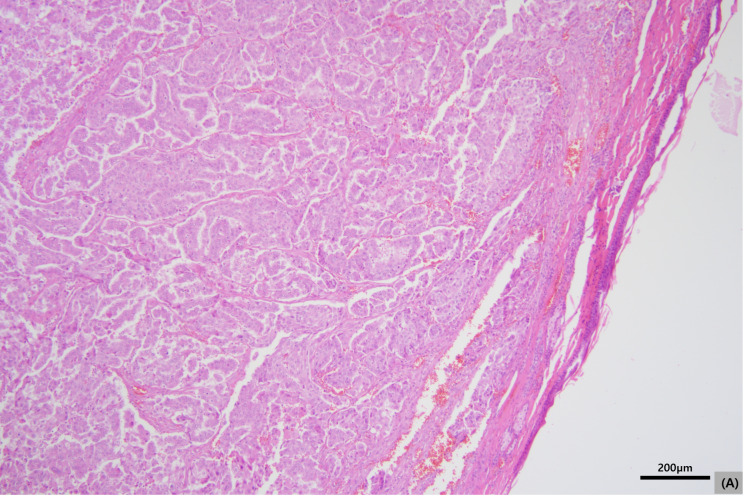

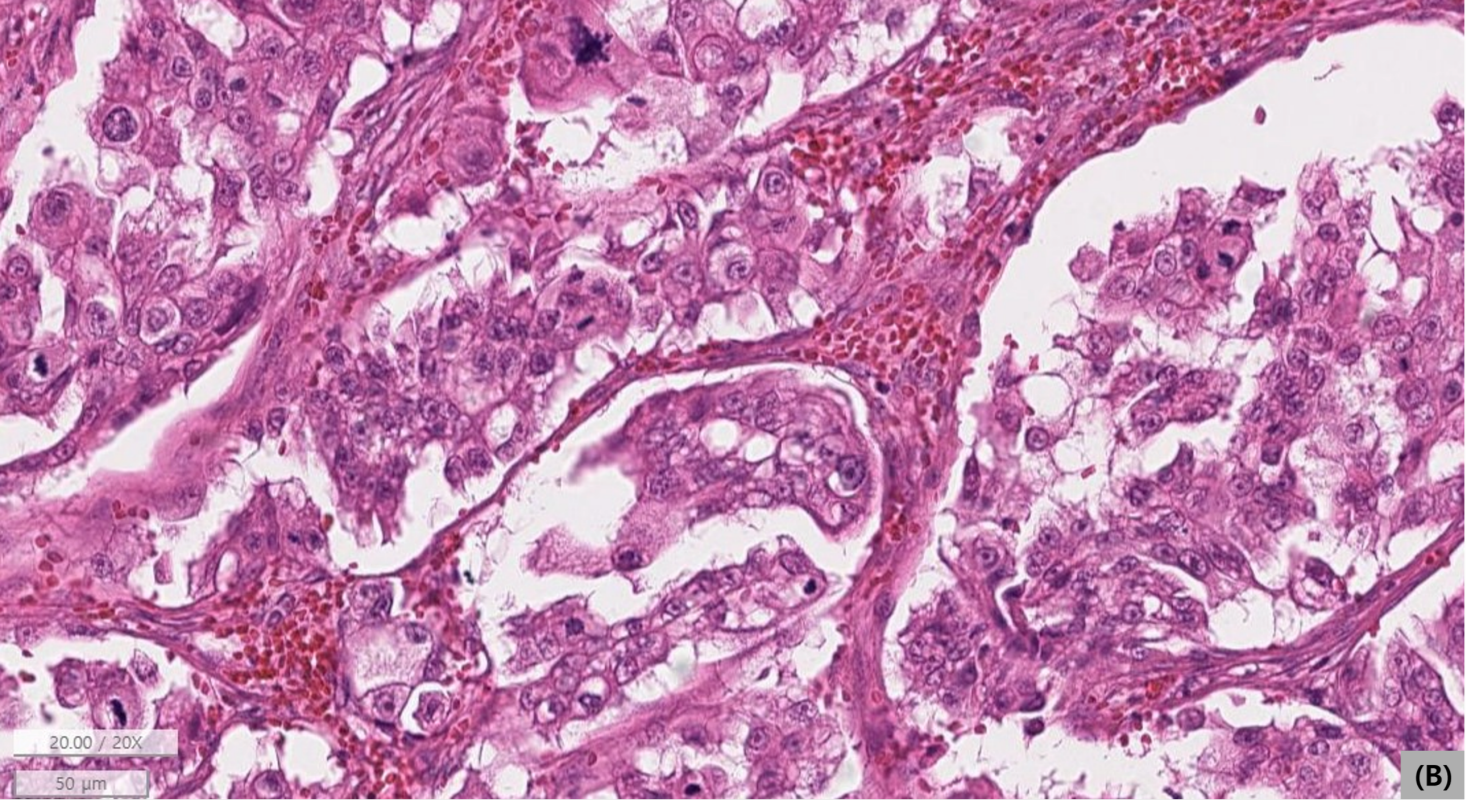

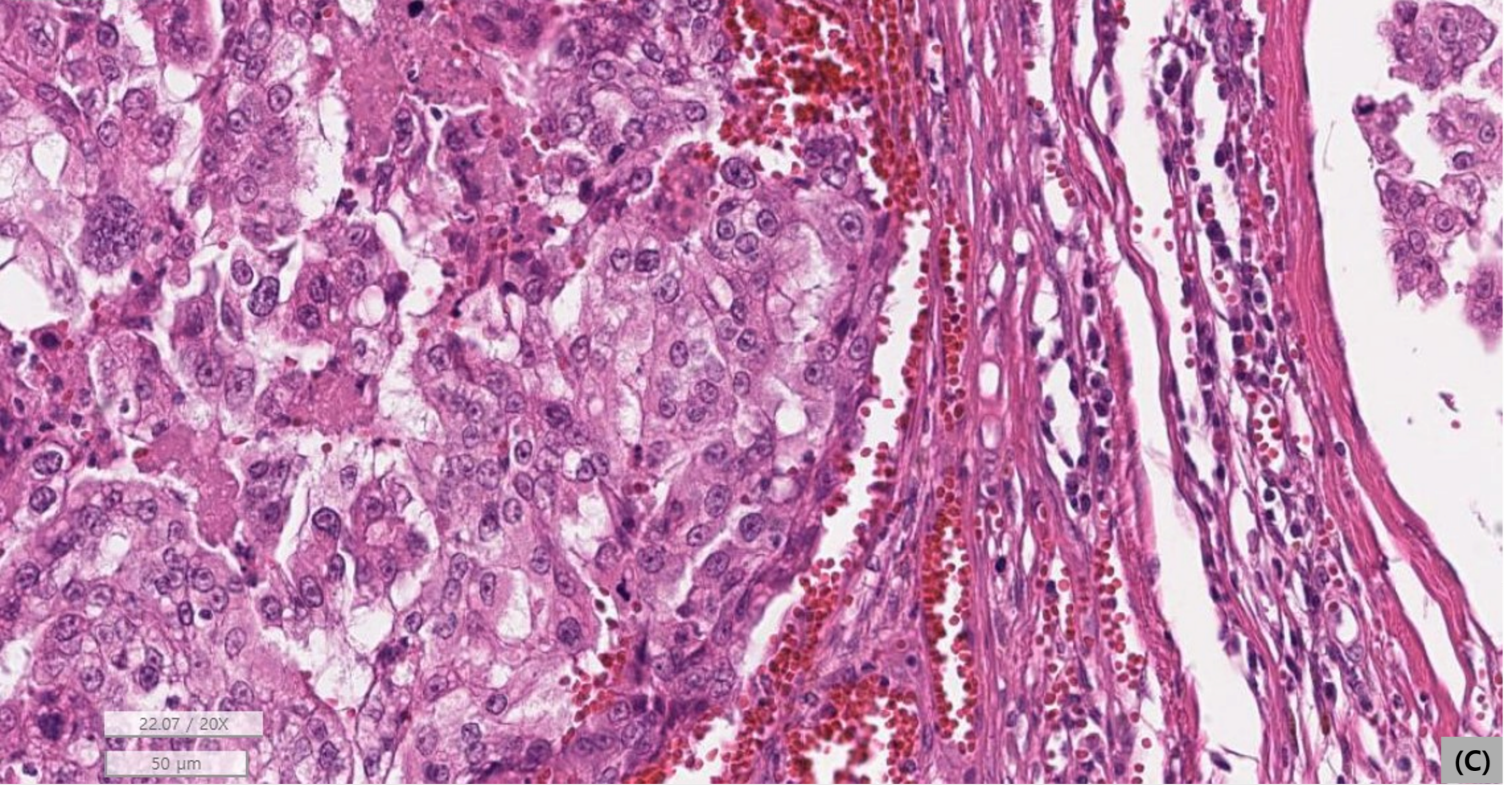

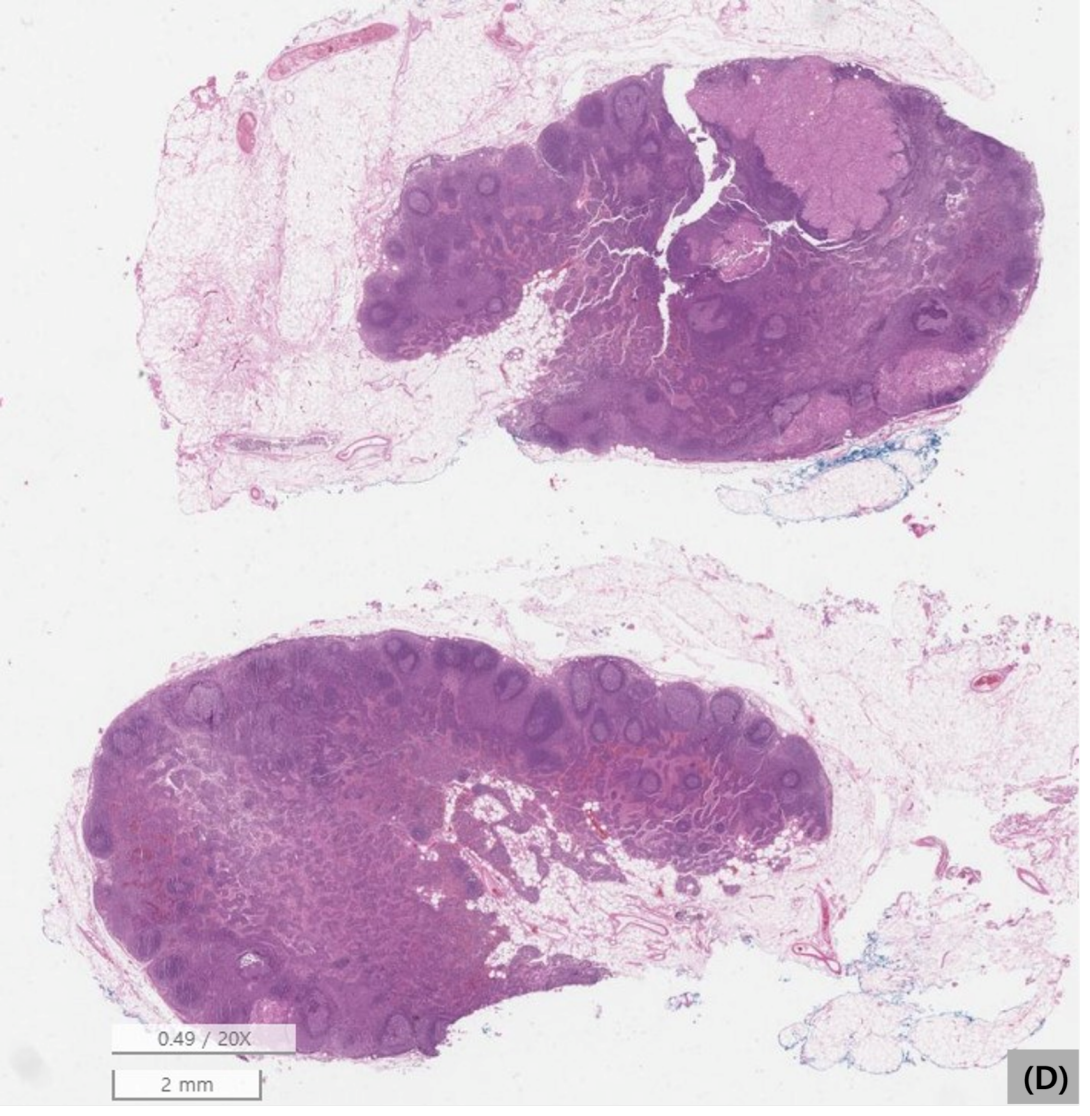

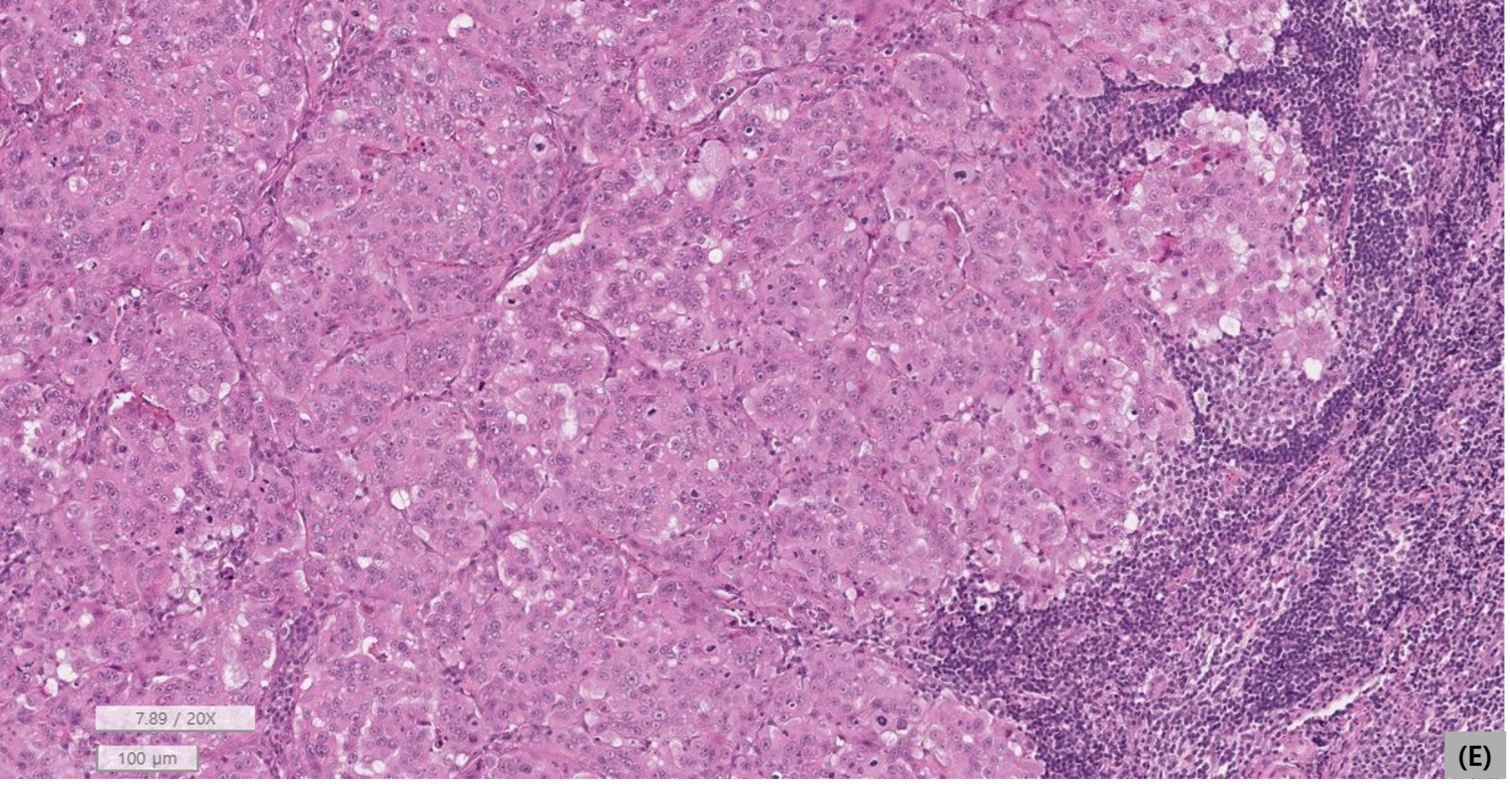

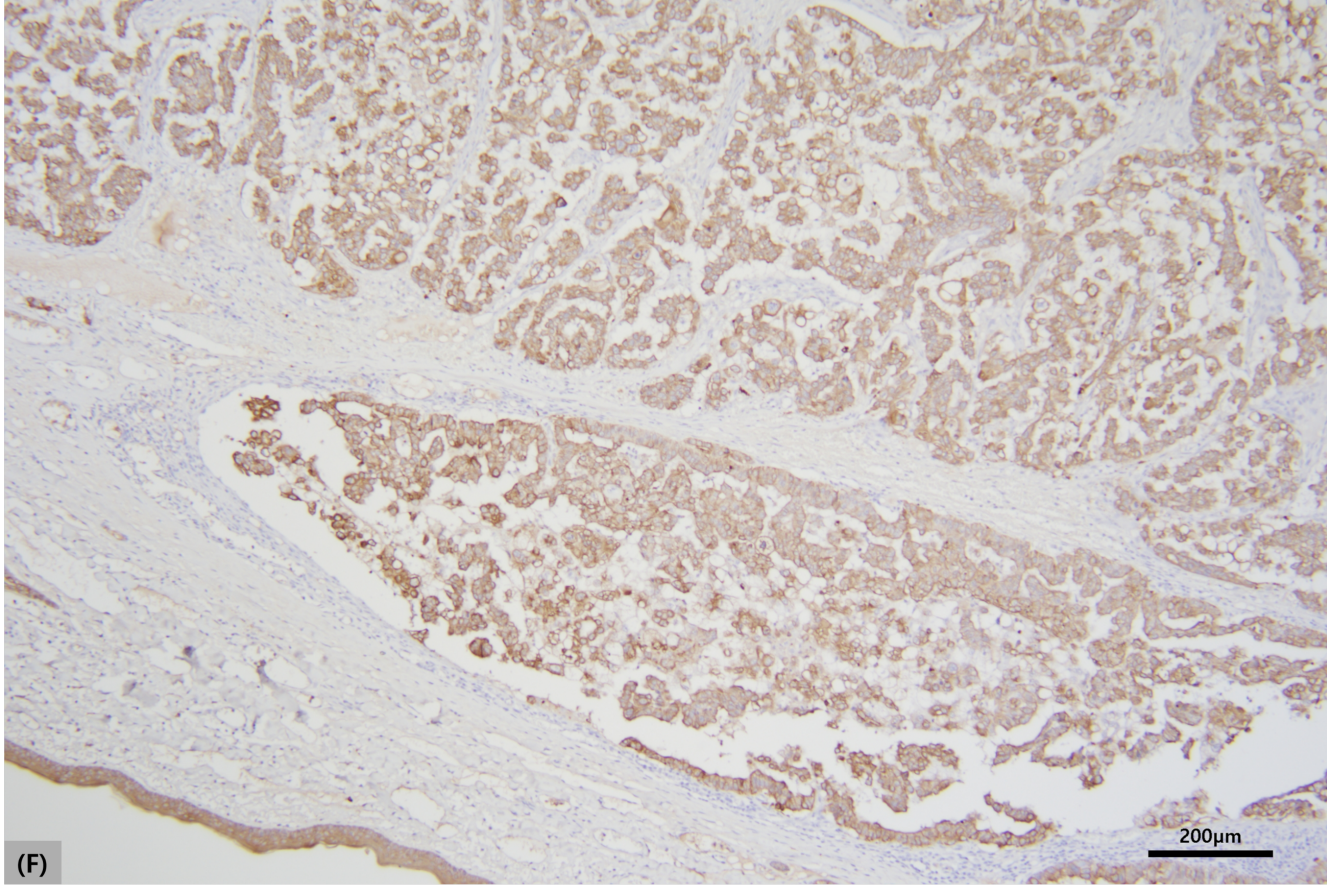

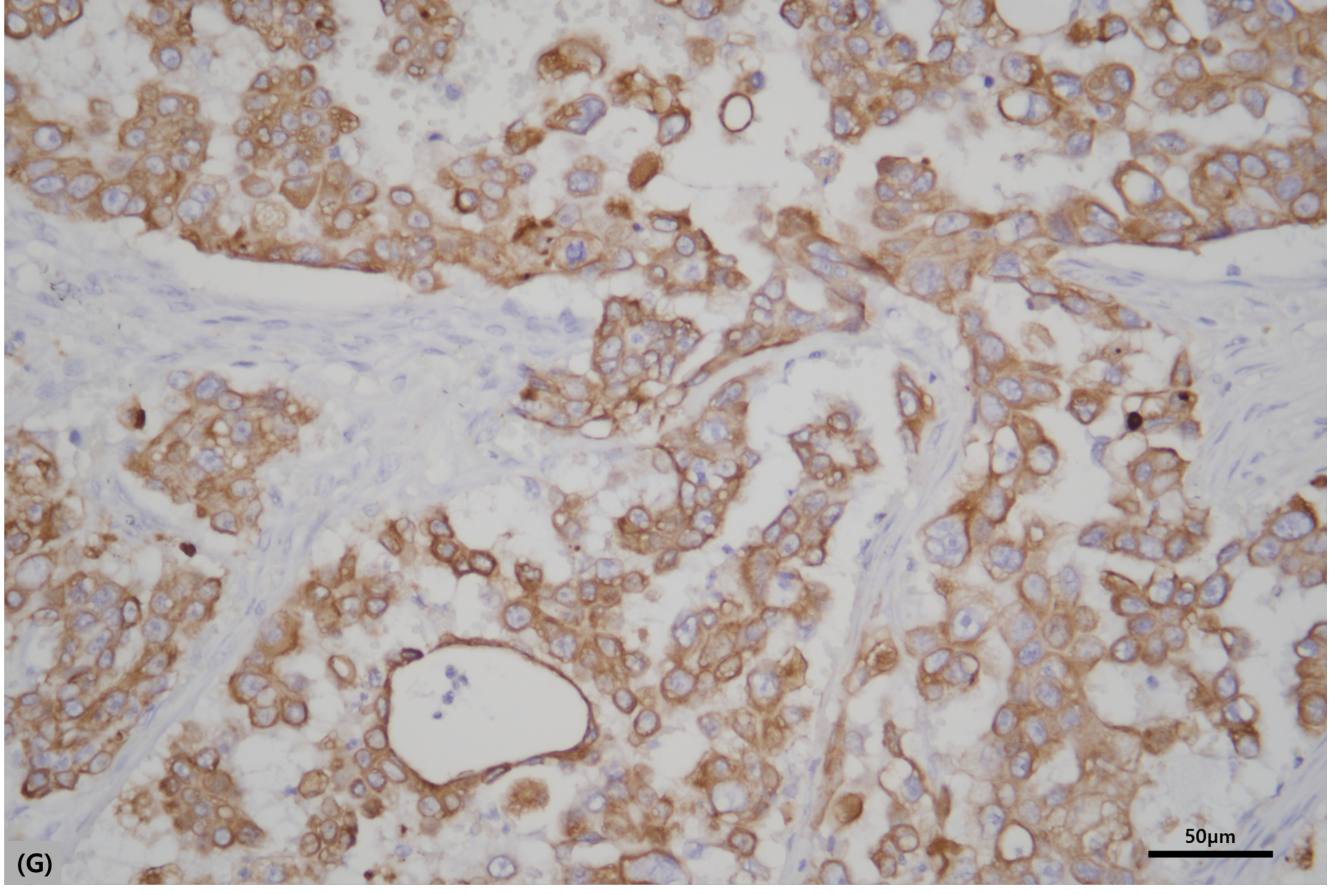
Histopathologic and pan-cytokeratin immunohistochemical findings of the primary tumor and metastatic ipsilateral mandibular lymph node. (**A**) Low-power view of the excised primary lower-lip mass, showing a relatively well-demarcated but unencapsulated epithelial neoplasm within the lip skin/subcutis (scale bar = 2 mm). (**B**) Higher-magnification view of the primary tumor, showing tubular/glandular proliferation of pleomorphic epithelial cells (scale bar = 50 µm). (**C**) Peripheral tumor cell nests adjacent to lymphovascular spaces; lymphovascular tumor emboli were identified histologically at the tumor periphery (scale bar = 50 µm). (**D**) Low-power view of the excised mandibular lymph node showing metastatic replacement of the cortex (scale bar = 2 mm). (**E**) Higher-magnification view of metastatic adenocarcinoma within the lymph node (scale bar = 100 µm). (**F**) Low- to intermediate-power pan-cytokeratin immunohistochemistry of the primary tumor showing cytokeratin-positive neoplastic epithelial cells (scale bar = 200 µm). (**G**) Higher-magnification pan-cytokeratin immunohistochemistry highlighting cytokeratin-positive neoplastic epithelial cells within tubular/glandular tumor structures (scale bar = 50 µm).

Microscopically, the primary tumor was centered within the superficial lip skin/subcutis and formed a relatively well-demarcated but unencapsulated proliferation of glandular epithelial cells arranged predominantly in tubular structures. The neoplastic cells showed marked pleomorphism, severe anisocytosis and anisokaryosis, multifocal necrosis and hemorrhage, and a mitotic count exceeding 30 per 10 high-power fields. Multiple tumor cell emboli were identified within lymphovascular channels at the tumor periphery. The measured tumor diameter on section was approximately 18.3 mm. Surgical margins were narrow but histologically complete, with the closest evaluable margin measuring at least 0.31 mm (Figure 5A–C).

Histopathology of the excised mandibular lymph node demonstrated metastatic adenocarcinoma morphologically similar to the primary lesion, with replacement of approximately 25% of the lymph node cortex by metastatic tumor (Figure 5D,E). Subsequent pan-cytokeratin immunohistochemistry was performed retrospectively on archived formalin-fixed, paraffin-embedded diagnostic tissue from the primary lower-lip mass using a mouse monoclonal anti-pan-cytokeratin antibody (clone AE1/AE3, M3515; DakoCytomation; dilution 1:200; primary antibody incubation at 4 °C overnight). Antigen retrieval was performed by microwave boiling in citric acid buffer (pH 6.0) for 10 min. Binding of the primary antibody was detected using the EnVision system-HRP (DakoCytomation, Carpinteria, CA, USA), with 3,3′-diaminobenzidine (DAB) as the chromogen. Internal mucosal epithelium served as an internal positive control. Immunoreactivity was assessed as cytoplasmic staining in neoplastic cells. Neoplastic cells showed positive immunoreactivity for pan-cytokeratin, supporting epithelial differentiation (Figure 5F,G). Periodic acid–Schiff (PAS) and Alcian blue histochemical staining of archived tumor tissue did not demonstrate distinct intratumoral mucin/mucopolysaccharide material, as evidenced by the absence of clear PAS-positive or Alcian blue-positive material within the tumor parenchyma. This finding did not support a mucin-rich salivary-type secretory phenotype and was concordant with the superficial cutaneous location, association with cutaneous adnexal structures, absence of histologic continuity with nearby minor salivary glands, and tubular/glandular histomorphology supporting a cutaneous adnexal/apocrine origin. Taken together, the final diagnosis was lower-lip ASGAC with lymphatic invasion and ipsilateral mandibular nodal metastasis.

Local healing progressed through POD 35, at which time the surgical site had contracted and no enlargement of the remaining palpable regional lymph nodes was appreciated. After discharge, structured oncologic follow-up, adjuvant treatment, and postoperative advanced restaging were not documented before the terminal event. On POD 87, the cat was re-presented on an emergency basis with severe anorexia, agonal breathing, and marked icterus, and cardiopulmonary resuscitation was unsuccessful. Terminal hematology, serum biochemistry, diagnostic imaging, and necropsy were not available; therefore, hepatobiliary disease, metastatic disease, paraneoplastic disease, systemic illness, or unrelated causes of icterus could not be distinguished, and a direct relationship between death and the carcinoma could not be established.

## 3. Discussion

This case illustrates the diagnostic risk of underestimating a hemorrhagic, superficially eroded same-site lower-lip lesion when cytology is nondiagnostic and objective resolution is not documented. The earlier lesion was not histologically characterized, and therefore, it cannot be proven to have represented the same neoplastic process as the later carcinoma. However, the combination of a same-site presentation, surface erosion/ulceration, bloody nondiagnostic aspiration, lack of tissue diagnosis, and absence of documented complete resolution supports a cautious interpretation of the clinical chronology. In such cases, repeat sampling or biopsy should be considered when a hemorrhagic or cystic-appearing lower-lip lesion persists or is subsequently noted at the same site.

Accurate assignment of tissue origin is a key diagnostic issue for malignant glandular tumors of the feline lower lip because both cutaneous adnexal and minor salivary glandular structures may be present in this region. In the present case, the diagnosis of ASGAC was supported by anatomic topography, histomorphology, histochemical findings, and epithelial immunoreactivity. The tumor was centered in the superficial lip skin/subcutis and was associated with adjacent cutaneous adnexal structures, including sebaceous glands, tactile hair follicles, and sweat glands. Nearby minor salivary gland tissue was present in the mucosal-side stroma, but it was separated from the neoplastic region by skeletal muscle, and no histologic continuity with the tumor was identified. The tubular/glandular architecture and pan-cytokeratin immunoreactivity supported epithelial glandular differentiation. Although distinct apical blebbing/decapitation secretion was not clearly identified, the tumor was poorly differentiated and markedly atypical, limiting reliance on this single morphologic feature. PAS and Alcian blue staining did not reveal distinct intratumoral mucin/mucopolysaccharide material and therefore did not support a mucin-rich salivary-type secretory phenotype. These histochemical stains were interpreted as ancillary findings rather than definitive markers of tissue origin. Taken together, the topographic, histomorphologic, histochemical, and immunohistochemical findings favored a cutaneous adnexal/apocrine origin over a minor salivary gland carcinoma, although salivary gland adenocarcinoma and salivary duct carcinoma remain important anatomic differentials in this location.

This case also demonstrates the value of tissue-based regional staging. CT delineated the local extent of the lower-lip mass, showed no obvious mandibular bone invasion, and did not identify distant metastasis. The ipsilateral mandibular lymph node was only mildly thickened and contrast-enhancing on CT, and the imaging interpretation remained indeterminate. Histopathology, however, confirmed metastatic replacement of approximately 25% of the excised mandibular lymph node cortex and identified lymphovascular tumor emboli in the primary tumor. Although extrapolation from canine head and neck tumors to feline lower-lip tumors should be made cautiously, these findings support histologic assessment of regional lymph nodes when malignancy is suspected in feline lower-lip glandular epithelial tumors [17,18,19,20].

The biological and prognostic conclusions that can be drawn from this single case are limited. The tumor showed marked pleomorphism, high mitotic activity, lymphovascular tumor emboli, lymphatic invasion, and histologically confirmed nodal metastasis, supporting aggressive biological behavior despite its modest clinical size. However, structured oncologic follow-up, adjuvant treatment, postoperative advanced restaging, terminal diagnostic testing, and necropsy were not available. The POD 87 presentation with severe icterus could have reflected hepatobiliary disease, metastatic disease, paraneoplastic disease, systemic illness, or an unrelated condition; therefore, death should not be interpreted as a confirmed tumor-related outcome. Despite these limitations, this case documents a rare feline lower-lip ASGAC with mandibular nodal metastasis and emphasizes the diagnostic importance of same-site lesion chronology, tissue-based diagnosis, and regional lymph node assessment.

## 4. Conclusions

This case documents feline lower-lip ASGAC with lymphatic invasion and mandibular nodal metastasis, diagnosed using integrated topographic, histomorphologic, histochemical, and immunohistochemical findings. The tumor’s superficial cutaneous location, association with cutaneous adnexal structures, lack of histologic continuity with nearby minor salivary glands, epithelial immunoreactivity, and ancillary histochemical findings collectively supported a cutaneous adnexal/apocrine origin. The earlier same-site hemorrhagic and superficially eroded lesion was not followed by scheduled reassessment or tissue diagnosis, and complete resolution was not objectively documented. Feline lower-lip glandular epithelial lesions with hemorrhagic, erosive, persistent, or subsequent same-site clinical concern may therefore require definitive histopathology and regional lymph node assessment for accurate diagnosis and staging.

## Data Availability

The data presented in this study are available from the corresponding author upon reasonable request. Access is restricted to protect the privacy of the client-owned animal and owner-related medical records.

## Abbreviations

The following abbreviations are used in this manuscript:

ASGAC	apocrine sweat gland adenocarcinoma
CT	computed tomography
FNA	fine-needle aspiration
H&E	hematoxylin and eosin
IHC	immunohistochemistry
LN	lymph node
POD	postoperative day
